# Impact of Lipoprotein Lipase Gene Polymorphism, *S447X*, on Postprandial Triacylglycerol and Glucose Response to Sequential Meal Ingestion

**DOI:** 10.3390/ijms17030397

**Published:** 2016-03-18

**Authors:** Israa M. Shatwan, Anne-Marie Minihane, Christine M. Williams, Julie A. Lovegrove, Kim G. Jackson, Karani S. Vimaleswaran

**Affiliations:** 1 Hugh Sinclair Unit of Human Nutrition, Department of Food and Nutritional Sciences, University of Reading, Reading RG6 6AP, UK; i.m.a.shatwan@pgr.reading.ac.uk (I.M.S.); A.Minihane@uea.ac.uk (A.-M.M.); j.a.lovegrove@reading.ac.uk (J.A.L.); k.g.jackson@reading.ac.uk (K.G.J.); 2 Food and Nutrition Department, Faculty of Home Economics, King Abdulaziz University, Jeddah 21589, Saudi Arabia; 3 Department of Nutrition and Preventive Medicine, Norwich Medical School, University of East Anglia, Norwich NR4 7TJ, UK; 4 Institute for Cardiovascular and Metabolic Research (ICMR), University of Reading, Reading RG6 6AS, UK; c.m.williams@reading.ac.uk

**Keywords:** lipoprotein lipase, sequential meals, postprandial study, triacylglycerol, glucose

## Abstract

Lipoprotein lipase (LPL) is a key rate-limiting enzyme for the hydrolysis of triacylglycerol (TAG) in chylomicrons and very low-density lipoprotein. Given that postprandial assessment of lipoprotein metabolism may provide a more physiological perspective of disturbances in lipoprotein homeostasis compared to assessment in the fasting state, we have investigated the influence of two commonly studied *LPL* polymorphisms (rs320, *HindIII*; rs328, *S447X*) on postprandial lipaemia, in 261 participants using a standard sequential meal challenge. *S447* homozygotes had lower fasting HDL-C (*p* = 0.015) and a trend for higher fasting TAG (*p* = 0.057) concentrations relative to the *447X* allele carriers. In the postprandial state, there was an association of the *S447X* polymorphism with postprandial TAG and glucose, where *S447* homozygotes had 12% higher TAG area under the curve (AUC) (*p* = 0.037), 8.4% higher glucose-AUC (*p* = 0.006) and 22% higher glucose-incremental area under the curve (IAUC) (*p* = 0.042). A significant gene–gender interaction was observed for fasting TAG (*p* = 0.004), TAG-AUC (*P*_interaction_ = 0.004) and TAG-IAUC (*P*_interaction_ = 0.016), where associations were only evident in men. In conclusion, our study provides novel findings of an effect of *LPL S447X* polymorphism on the postprandial glucose and gender-specific impact of the polymorphism on fasting and postprandial TAG concentrations in response to sequential meal challenge in healthy participants.

## 1. Introduction

Large prospective cohort studies have identified elevated non-fasting triacylglycerol (TAG) concentrations as an independent risk factor for cardiovascular disease (CVD) [1]. The Copenhagen City Heart Study [2] and US Women’s Health Study [3] showed that non-fasting TAG concentrations of ≥5.0 mmol/L were associated with myocardial infarction and the Norwegian Counties Study [4] showed that non-fasting TAG levels of >3.5 mmol/L were associated with a 5-fold increase in risk of death from coronary heart disease. Endothelial associated Lipoprotein lipase (LPL) (E.C. 3.1.1.34) plays an important role in the metabolism and clearance of triacylglycerol (TAG)-rich lipoproteins from the circulation [5] and atherogenesis, where it influences the interaction between atherogenic lipoproteins and receptors on the vascular wall [6]. Hence, enzymes such as Lipoprotein lipase (LPL) (E.C. 3.1.1.34) that regulate lipoprotein metabolism in the postprandial state [7] are of interest to the prevention of CVDs.

Several polymorphisms in the *LPL* gene have been shown to lead to a reduction in enzyme synthesis and activity and, to date, rs320 [*HindIII* (G/T)] and rs328 [*Serine 447 Stop S447X* (C/G)] have been the most extensively studied. These variants with a prevalence of 40%–75% (rs320) and 17%–22% (rs328) among Caucasians [8,9], respectively, have been shown to be associated with coronary artery disease, myocardial infarction [10,11,12] and pronounced fasting hypertriacylglycerolemia [13,14]. Only limited number of studies has examined their impact on postprandial lipaemia [15,16,17] and these studies have used only a single test meal, which does not reflect the habitual eating pattern in humans.

Given that we spend nearly 75% of the time in a postprandial state, the normal physiological pattern of meal intake and the impact of *LPL* gene polymorphisms on the clearance of dietary TAG may be more evident after a sequential meal challenge. Hence, in the present study, we investigated the association of the two commonly studied *LPL* polymorphisms [rs320 (*HindIII*) and rs328 (*S447*)] with fasting and postprandial lipid concentrations by using a standard sequential meal challenge and examined the penetrance of genotype according to gender, with gender previously shown to be a modulator of the impact of other variants on postprandial TAG handling [18].

## 2. Results

The study participants included 153 men (mean ± SD, age 53 ± 10 years; BMI 27.3 ± 3.1 kg/m^2^) and 109 women (mean ± SD, 52 ± 11 years; BMI 25.4 ± 3.5 kg/m^2^). The prevalence of *S447* homozygotes was 81% (*n* = 213) *versus* 18% *447X* minor allele carriers (*n* = 48). The frequency of carriers of *447X* in this study was consistent with published reports in Caucasian population [19]. 56% of participants were homozygous for the H1 major allele for *HindIII* (*n* = 131) with 43% H2 minor allele carriers (*n* = 100).

Table 1 describes the baseline characteristics of participants according to the *S447X* polymorphism. There was a borderline genotype-related association with fasting serum TAG levels after adjusting for age, gender, and BMI (*p* = 0.057). Circulating HDL-C concentrations were markedly lower in *S447* homozygotes than *447X* allele carriers (*p* = 0.015). None of the other fasting biochemical parameters were significantly associated with *S447X* (Table 1) or *HindIII* polymorphisms (Supplementary Table S1) (all *p* > 0.05).

A greater TAG AUC was observed in *S447* allele homozygotes following the sequential meals (*p* = 0.037). The area under the curve (AUC) and incremental area under the curve (IAUC) of the glucose response was 8.4% (*p* = 0.006) and 22.6% (*p* = 0.042) lower in *447X* allele carriers (*p* = 0.006), respectively than common homozygotes. The postprandial summary measures did not show any association with *HindIII* genotypes (Supplementary Table S1).

**Figure 1:**
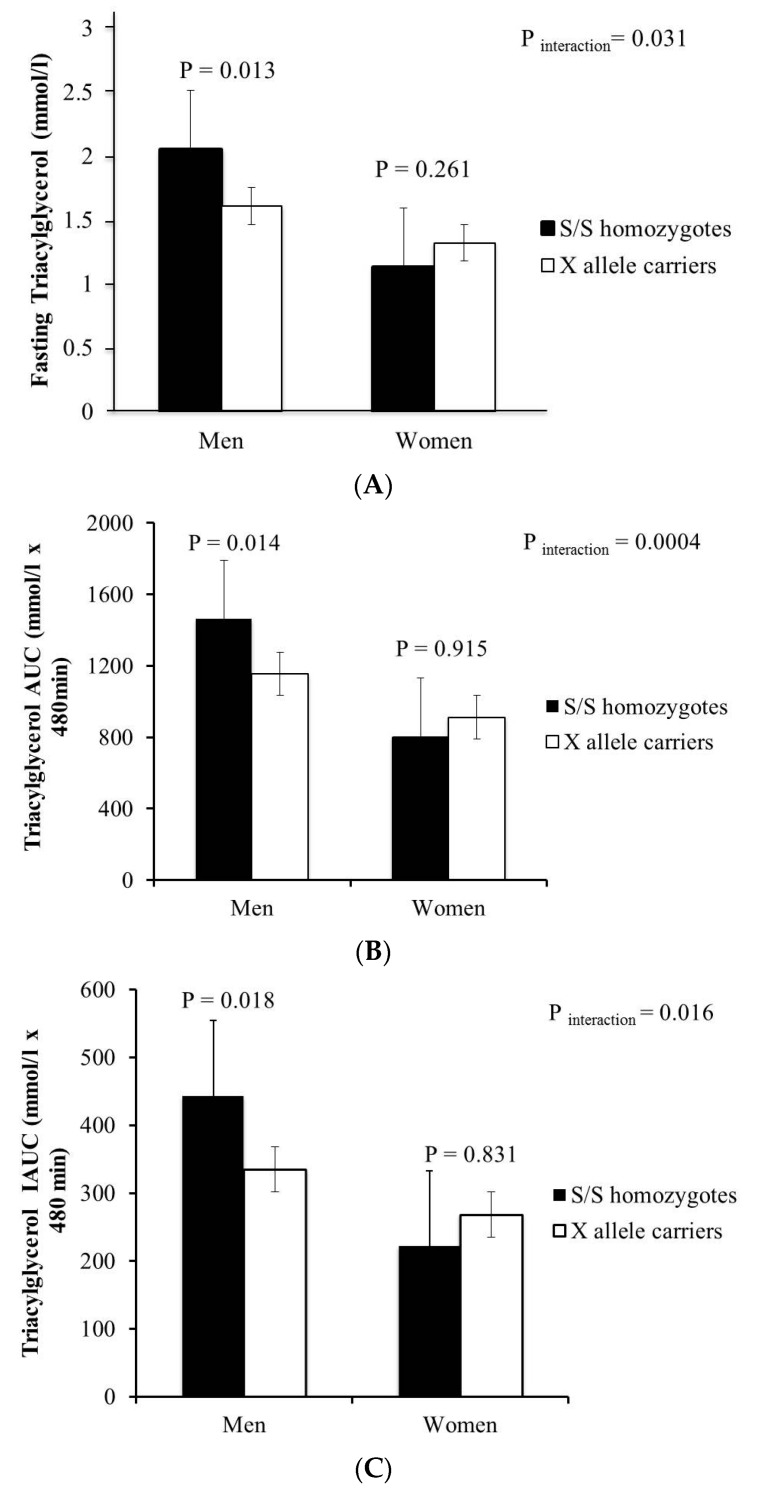
(**A**) Mean (SEM) for fasting triacylglycerol (TAG) according to *S447X* polymorphism in men and women. Carriers of one or two copies of X minor allele are combined and presented by white bars. Gene–gender interaction was statistically significant for fasting TAG levels (*P*_interaction_ = 0.031); (**B**) Mean (SEM) for the area under the curve (AUC) TAG response according to *S447X* polymorphism after consumption of a test breakfast (49 g fat) at 0 min and a test lunch (29 g fat) at 330 min. *S447* homozygotes (*n* = 213) had 12% higher TAG AUC (*p* = 0.037) compared to *447X* carriers (*n* = 48) for men. Carriers of one or two copies of X minor allele are combined and presented by white bars. Gene–Gender interaction was statistically significant for area under the TAG curve values (*P*_interaction_ = 0.004); (**C**) Mean (SEM) for the incremental area under the curve (IAUC) TAG response according to *S447X* polymorphism after consumption of a test breakfast (49 g fat) at 0 min and a test lunch (29 g fat) at 330 min in men, and women. Carriers of one or two copies of X minor allele are combined and presented by white bars. Gene–Gender interaction was statistically significant for IAUC TAG (*P*_interaction_ = 0.016).

The *S447X* polymorphism showed a significant interaction with gender on fasting TAG (*p* = 0.004), TAG AUC (*p* = 0.004) and TAG IAUC (*p* = 0.016) (Figure 1), with the major *S447* allele homozygotes at *S447X* in men showing higher values for fasting and postprandial TAG compared with X minor allele carriers.

Given the strong linkage disequilibrium between the two *LPL* polymorphisms [15], we also examined the combined effects of the polymorphisms on baseline characteristics and postprandial TAG and glucose. Nine possible genotype combinations were generated. However, given the small sample size, only 3 combinations [*S447S* − H1/H1 (*n* = 131), *S447S* − H1/H2 (*n* = 55) and *S447X* − H1/H2 (*n* = 45)] were available in our study participants. The frequencies of these three genotype combinations are presented in the Supplementary Table S2. In the genotype-genotype analysis, we found that individuals with the *S447S* genotype irrespective of HindIII alleles (*i.e.*, SS/H1H1 and SS/ H1H2) had higher TAG AUC (*p* = 0.040) and glucose AUC (*p* = 0.034) levels than *447X* allele carriers (Supplementary Table S3) suggesting that the associations are driven mainly by the *S447X* polymorphism.

## 3. Discussion

Our postprandial study using a standard sequential meal challenge demonstrates that individuals homozygous for the common allele of the *S447X* polymorphism had significantly lower fasting HDL-C levels and a significantly elevated postprandial TAG and glucose response relative to *447X* allele carriers. In addition, a gender-specific association between the *S447X* polymorphism and fasting and postprandial TAG concentrations was observed, where the effect of the genotype was evident only in men.

Several studies have demonstrated the association between *S447* genotype and elevated fasting plasma TAG levels and lower HDL-C levels [20,21]. Our study also has shown a borderline association of the *S447* allele with higher TAG and a significant association with lower HDL-C levels, which is in accordance with a meta-analysis (*n* = 45,079) that showed 0.05 mmol/L lower HDL-C levels and 0.15 mmol/L higher TAG among the *S447* homozygotes [12]. Furthermore, the association of the *S447* genotype with postprandial TAG in our study has also been confirmed in previous studies (Supplementary Table S4). However, the mechanism by which this polymorphism affects lipid levels still remains obscure. Functional studies have demonstrated that the *447X* variant that results in a 2 amino acid truncation on the carboxyl-terminal domain of the LPL increases the ability of the cell surface receptors to bind with TAG-lipoproteins [8]; but it is not clear how this truncation increases the ability of LPL to bind TAG. While a few studies have reported that *S447X* polymorphism might increase or decrease the LPL activity [22,23,24], some have failed to show a significant effect [23,25]. The probable mechanism by which *LPL S447* allele lowers HDL-C could be related to higher TAG concentrations. A delayed clearance of triglyceride-rich lipoproteins (TRLs) drives the transfer of TAG from TRLs to both LDL and HDL by cholesteryl ester transfer protein (CETP), which makes them suitable substrates for lipases. This leads to the formation of smaller denser HDL particles which are rapidly removed from the circulation, thus decreasing HDL-C concentrations [26].

We also observed a novel association between *LPL S447X* polymorphism and postprandial glucose using the sequential meal challenge, where *S447* homozygotes had higher glucose AUC and IAUC. Previous postprandial studies have shown higher postprandial glucose concentrations in response to three meals [27,28]. Given that the postprandial studies examining the effects of *LPL* polymorphisms have used only a single meal [15,16,17], it is possible that the effects of *LPL* polymorphisms on postprandial glucose have been missed previously. One of the reasons for higher postprandial glucose could be due to the decreased insulin sensitivity in the *S447* homozygotes [29]. LPL has been considered as a link between insulin resistance and atherosclerosis, given its role in controlling the delivery of free fatty acids to muscle, adipose tissue and vascular wall macrophages, wherein lipid uptake influences insulin sensitivity [30]. It was also shown in the Quebec Family study that the *LPL* markers (*HindIII* and *S447X*) combination influenced the insulin AUC during an oral glucose tolerance test [31]. In our study, we did not find any association with fasting and postprandial insulin and HOMA-IR, which might be due to limited power for this analysis as insulin concentrations were not available for all participants (*n* < 166). However, given that low HDL cholesterol and high TAG are frequently found with insulin resistance [32], it is possible that decreased insulin sensitivity could be a possible mechanism for higher postprandial glucose concentrations in the *S447* homozygotes. This was also shown in a previous study where individuals with insulin resistance had elevated fasting and postprandial TAG, and lower HDL-C levels [33].

Our results also demonstrated gender-specific effect of *LPL S447X* polymorphism on fasting and postprandial TAG, where the association was significant only in men. The gender-specific effects of other SNPs (*LEPR* [34], *APOA5* [18] and *APOB* [35]) in men have already been shown in our postprandial cohort which is characteristic of men with higher BMI, fasting TAG, insulin and lower HDL-C than women irrespective of the genotype. A postprandial study in 63 men also showed that those with low fasting HDL-C and high TAG concentrations had higher postprandial TAG [36]. It is possible that men from our cohort were at a greater metabolic stress which may be a contributory factor in the gender-specific effect of *S447* genotype on the fasting and postprandial responses.

Previous studies have shown associations between the *HindIII* polymorphism and elevated lipids [10]. In this study, we did not find any significant difference in fasting and postprandial lipid levels across the genotypes of the polymorphism. However, the combination of *HindIII* and *S447X* markers revealed significant associations with TAG and glucose AUC, which might be due to the strong LD between the *HindIII* and *S447X* markers. In addition, previous studies have investigated the effect of this polymorphism on postprandial lipids using only a single meal [15]. Hence, our finding with HindIII polymorphism requires a replication using a sequential meal challenge, which reflects the habitual eating pattern.

In conclusion, our study provides novel findings of an effect of *LPL S447X* polymorphism on the postprandial glucose and gender-specific impact of the polymorphism on fasting and postprandial TAG levels in response to sequential meal challenge in healthy participants. The elevated fasting and postprandial TAG and postprandial glucose and lower fasting HDL-C concentrations are likely to result in prolonged appearance of lipids, in particular remnant particles, which might be one of the reasons for the increased prevalence of CVD in participants carrying the *S447* allele. Further studies are required to confirm our gender-specific associations between the *LPL* polymorphism and fasting and postprandial TAG levels with an assessment of serum LPL concentrations and activity using a sequential meal challenge. This will further shed light on the size-effect of *LPL* polymorphisms on lipid and glucose metabolism in population subgroups.

## 4. Experimental Section

### 4.1. Subjects

All individuals included in this study were obtained from postprandial studies using identical inclusion/exclusion criteria, and all underwent the same sequential meal postprandial protocol, at University of Reading between 1997 and 2007, as previously described [37]. Briefly, 261 participants (109 women and 153 men) aged 22–71 years and BMI 17.6–37.3 kg/m^2^, were included in the dataset. The postprandial study excluded participants who had CVD, including angina stroke; diabetes or fasting glucose > 6.5 mmol/L, liver or other endocrine dysfunction; were pregnant or lactating; who were smoking > 15 cigarette per day; doing aerobic exercise more than three times per week; who had hemoglobin levels < 130g/L for men and 120g/L for women or taking medication or supplements. The University of Reading Ethics and Research Committee and the West Berkshire Health Authority Ethics Committees approved the experimental protocol. Informed consent to participate in the study was obtained.

### 4.2. Sequential Test Meal Protocols

Details of the postprandial protocol have been described previously [37]. Briefly, study participants were asked to refrain from alcohol or organised exercise regimens on the previous day and were provided with a relatively low fat (<10 g fat) evening meal to standardise short-term fat intake. After a 12 h overnight fast, the participants were cannulated and fasting blood sample was taken. Following a standard test breakfast (0 min; 3.9 MJ energy, 111 g carbohydrate, 19 g protein and 49 g fat) and lunch (330 min; 2.3 MJ energy, 63 g carbohydrate, 15 g protein and 29 g fat), blood samples were taken from the cannula at 30–60 min intervals until 480 min after the test breakfast.

### 4.3. Biochemical Measurements

Plasma glucose and lipid concentrations were measured using an automated analyzer assay (Instrumentation Laboratory (UK) Ltd, Warrington, UK). In the fasting sample, HDL-C was estimated in the supernatant following precipitation of the apolipoprotein B (apoB)-containing lipoproteins with a dextran-manganese chloride reagent. The LDL-C concentration was calculated using the Friedewald formula. Insulin levels were determined by ELISA (Dako Ltd, High Wycombe, UK). The total area under the curve (AUC, 0–480 min) was calculated using the trapezium rule, and incremental area under the curve (IAUC) calculated by subtracting the fasting levels from the total AUC. For NEFA, AUC and IAUC were calculated from the time of suppression until the end of the postprandial period (120–480 min) due to initial drop in NEFA concentrations after the meal. The homeostasis model assessment of insulin resistance (HOMA-IR) was calculated using formula: [fasting insulin (pmol/L) × fasting glucose (mmol/L)]/135.

### 4.4. DNA Extraction and Genotyping

DNA was isolated from the buffy coat layer of 10 mL of EDTA blood using the Qiagen DNA Blood Mini Kit (Qiagen Ltd, Crawley, UK). Allelic discrimination of two *LPL* gene polymorphisms (rs320, *HindIII* and rs328, *S447*) was conducted using a “Assay-on-Demand” SNP genotyping assays (Applied Biosystems, Warrington, UK).

### 4.5. Statistical Analysis

The statistical significance of associations between biochemical data and genotypes was established using a general linear model adjusted for covariates such as age, gender, and BMI, which are highly correlated to lipid concentrations. Genotype distribution for *LPL* SNPs was assessed using the Hardy-Weinberg equilibrium. Given the small number of rare homozygotes, we applied a dominant model in which carriers of 1 or 2 copies of the minor allele of the two polymorphisms *HindIII* and *S447X* were grouped and compared with major allele homozygotes. Interaction between gender and polymorphisms on outcomes was examined by introducing the interaction terms into the linear regression analysis models with adjustment to same variables in association test. All data presented in the text and tables represents mean ± SD. We used SPSS software (version 21; SPSS Inc, Chicago, IL, USA) for all statistical analyses. Probability values under 0.05 were considered significant.

## Figures and Tables

**Table 1:** Baseline and postprandial characteristics of the participants according to *LPL-S447X* polymorphism.

Participant’s Characteristics	S/S (*n* = 213)	S/X (*n* = 48)	*P*_association_
Age (years)	53 ± 11	52 ± 11	0.648
Men/Women	125/88	27/21	–
BMI (kg/m^2^)	26.3 ± 3.4	27.4 ± 3.2	0.015
**Baseline Characteristics**			
TC (mmol/L)	5.78 ± 1.05	5.62 ± 1.00	0.285
TAG (mmol/L)	1.67 ± 0.90	1.48 ± 0.51	0.057
HDL-C (mmol/L)	1.29 ± 0.42	1.40 ± 0.33	0.015
LDL-C (mmol/L)	3.73 ± 1.02	3.53 ± 0.92	0.167
Glucose(mmol/L)	5.16 ± 0.66	5.14 ± 0.45	0.534
Insulin (pmol/L)	48.3 ± 31.2	50.3 ± 26.2	0.517
NEFA (μmol/L)	519 ± 184	477 ± 170	0.102
HOMA-IR	1.96 ± 1.40	1.99 ± 1.09	0.376
**Postprandial summary measures**			
TAG AUC (mmol/L × 480 min)	1193 ± 593	1046 ± 429	0.037
TAG IAUC (mmol/L × 480 min)	353 ± 228	305 ± 210	0.149
NEFA AUC mmol/L × 300 min	153 ± 45	149 ± 32	0.410
NEFA IAUC (mmol/L × 300 min)	96 ± 39	100 ± 26	0.792
Glucose AUC (mmol/L × 480 min)	3114 ± 460	2850 ± 763	0.006
Glucose IAUC (mmol/L × 480 min)	595 ± 284	460 ± 238	0.042
Insulin AUC (nmol/L × 480 min)	139 ± 94	123 ± 38	0.858
Insulin IAUC (nmol/L × 480 min)	114 ± 89	100 ± 34	0.876

Values are mean ± standard deviation. *p*-Values are from a linear model testing the association with *LPL*-*S447X*, adjusted for age, gender, BMI; Abbreviations: TC, total cholesterol; TAG, triacylglycerol; HDL-C, high density lipoprotein cholesterol; LDL-C, low density lipoprotein cholesterol; NEFA, non-esterified fatty acids; HOMA-IR, homeostasis model assessment—insulin resistance; For the baseline analysis, the insulin and HOMA-IR values were available for 166 participants (men = 124, women = 42); For the postprandial analysis, the insulin AUC and IAUC data was available for 79 participants (men = 68, women = 11).

## Supplementary Materials

Supplementary materials can be found at http://www.mdpi.com/1422-0067/17/3/397/s1.

## Abbreviations

LPL: lipoprotein lipase; BMI: body mass index; CVD, cardiovascular disease; TAG: triacylglycerol; TC, total cholesterol; HDL-C: high-density lipoprotein cholesterol; LDL-C: low-density lipoprotein cholesterol; VLDL: very-low-density lipoprotein; NEFA: Non-esterified fatty acids; HOMA-IR: Homeostasis model assessment-insulin resistance; SNP: Single-nucleotide polymorphism; AUC: area under the curve; IAUC: incremental area under the curve.
