# Supplementary material for: Impact of Lipoprotein Lipase Gene Polymorphism, S447X, on Postprandial Triacylglycerol and Glucose Response to Sequential Meal Ingestion
Source: Int J Mol Sci. 2016 Mar 18;17(3):397. doi: 10.3390/ijms17030397 (PMC4813252; doi:10.3390/ijms17030397)
Supplement: Supplementary file 1 [file ijms-17-00397-s001.pdf]

Supplementary Materials: Impact of Lipoprotein Lipase Gene Polymorphism, *S447X*, on Postprandial Triacylglycerol and Glucose Response to Sequential Meal Ingestion

Israa M. Shatwan, Anne-Marie Minihane, Christine M. Williams, Julie A. Lovegrove, Kim G. Jackson and Karani S. Vimaleswaran

Table S1. Baseline and postprandial characteristics of participants according to the *LPL-HindIII* polymorphism.

Participant's Characteristics	*HindIII* rs320		*p* Value
	H1 (*n* = 131)	H1/H2 (*n* = 100)	
Age (years)	53 ±11	52 ±10	0.567
BMI (kg/m^2)	26.0 ±3.4	26.6 ±3.1	0.095
TC (mmol/L)	5.62 ±1.02	5.67 ±1	0.517
TAG (mmol/L)	1.53 ±0.87	1.55 ±0.65	0.972
HDL-C (mmol/L)	1.35 ±0.42	1.36 ±0.39	0.526
LDL-C (mmol/L)	3.59 ±0.96	3.59 ±0.98	0.872
Glucose(mmol/L)	5.14 ±0.69	5.10 ±0.50	0.413
Insulin (pmol/L)	45.9 ±30.0	52.2 ±31.9	0.414
NEFA (μmol/L)	530 ±200	498 ±173	0.177
HOMA-IR	1.87 ±1.36	2.06 ±1.35	0.680
TAG AUC (mmol/L × 480 min)	1086 ±549	1098 ±464	0.947
TAG IAUC (mmol/L × 480 min)	321 ± 212	335 ±230	0.677
NEFA AUC (mmol/L × 300 min)	155 ± 40	153 ± 49	0.600
NEFA IAUC (mmol/L × 300 min)	99 ± 33	94 ± 45	0.231
Glucose AUC (mmol/L × 480 min)	3109 ± 408	2958 ± 705	0.065
Glucose IAUC (mmol/L × 480 min)	590 ± 287	558 ± 269	0.648
Insulin AUC (nmol/L × 480 min)	128 ± 59	134 ± 130	0.782
Insulin IAUC (nmol/L × 480 min)	105 ± 54	109 ± 125	0.879

Values are mean ± standard deviation; *p*-Values are from a linear model testing the association with *LPL* genotypes, adjusted for age, gender, BMI; Abbreviations: TC, total cholesterol; TAG, triacylglycerol; HDL-C, high density lipoprotein cholesterol; LDL-C, low density lipoprotein cholesterol; NEFA, non-esterified fatty acids; HOMA-IR, homeostasis model assessment-insulin resistance, AUC, area under the curve; IAUC, incremental area under the curve; The fasting and postprandial insulin and HOMA-IR values were not available for all participants (*n* = 151).

Table S2. Distribution of study individuals according to HindIII and S447X Combined Genotypes.

S447X-HindIII	S/S	S/X	X/X
**H1/H1**	131	0	0
**H1/H2**	55	45	0
**H2/H2**	0	0	0

Table S3. Association between the combined genotypes of *LPL S447X-HindIII* polymorphisms and fasting and postprandial characteristics.

Participant's Characteristics	*S447X-HindIII*			*p* Value
	SS/H1 *n* = 131	SX/H1H2 *n* = 45	SS/H1H2 *n* = 55	
Age (years)	53 ± 11	52 ± 11	52 ± 9	0.570
BMI (kg/m²)	25.98 ± 3.44	27.24 ± 3.18	26.15 ± 2.93	0.052
TC (mmol/L)	5.62 ± 1.02	5.58 ± 0.99	5.75 ± 1.01	0.649
TAG (mmol/L)	1.53 ± 0.87	1.44 ± 0.47	1.64 ± 0.76	0.230
HDL-C (mmol/L)	1.35 ± 0.42	1.41 ± 0.33	1.32 ± 0.43	0.128
LDL-C (mmol/L)	3.59 ± 0.96	3.50 ± 0.91	3.67 ± 1.04	0.596
Glucose(mmol/L)	5.14 ± 0.69	5.12 ± 0.49	5.09 ± 0.53	0.708
Insulin (pmol/L)	45.9 ± 30.0	49.9 ± 26.6	54.18 ± 36	0.298
NEFA (µmol/L)	530 ± 200	471 ± 171	519 ± 1738	0.145
HOMA-IR	1.87 ± 1.36	1.97 ± 1.11	2.13 ± 1.54	0.434
TAG AUC (mmol/L × 480 min)	1086 ± 549	995 ± 369	1183 ± 518	0.040
IAUC (mmol/L × 480 min)	321 ± 212	302 ± 215	361 ± 241	0.262
NEFA AUC (mmol/L × 300 min)	155 ± 40	149 ± 33	156 ± 59	0.554
IAUC (mmol/L × 300 min)	99 ± 33	100 ± 27	89 ± 55	0.333
Glucose AUC (mmol/L × 480 min)	3109 ± 408	2831 ± 797	3057 ± 617	0.034
IAUC (mmol/L × 480 min)	590 ± 287	454 ± 248	636 ± 260	0.051
Insulin AUC (nmol/L × 480 min)	128 ± 60	119 ± 38	145 ± 169	0.948
IAUC (nmol/L × 480 min)	105 ± 54	95 ± 303	118 ± 164	0.967

Abbreviations: TC, total cholesterol; TAG, triacylglycerol; HDL-C, high density lipoprotein cholesterol; LDL-C, low density lipoprotein cholesterol; NEFA, non-esterified fatty acids; HOMA-IR, homeostasis model assessment-insulin resistance, AUC, area under the curve; IAUC, incremental area under the curve.

Table S4. List of postprandial studies determining the effects of *LPL* gene polymorphisms (*S447X* and *HindIII*) on fasting and postprandial lipids.

Reference	Sample Size	Meal Composition	*LPL* Polymorphism	Outcome	Association (*p* Value)
Lopez-Miranda *et al.* 2004 [1]	51	High fat meal (60 g fat and 420 mg cholesterol—60% fat, 15% protein, and 25% carbohydrates)	HindIII	Postprandial Small triacylglycerol-rich lipoproteins (TRL)-retinyl palmitate (RP)	$p = 0.030$
			S447X	Small TRL-RP<br>Large TRL-B48<br>Small TRL-B48	$p = 0.028$<br>$p = 0.046$<br>$p = 0.048$
			HindIII- S447X	Fasting Triacylglycerol (TAG)<br>Large TRL-TG<br>Large TRL-RP<br>Small TRL-RP<br>Large TRL-B48	$p = 0.047$<br>$p = 0.048$<br>$p = 0.004$<br>$p = 0.014$<br>$p = 0.036$
Humphries *et al.* 1998 [2]	2181	Oral liquid lipid load (42 g saturated fat, 22 g protein, 56 g carbohydrate, and 417 mg cholesterol)	*H-/X*447	Fasting TAG<br>–<br>Postprandial TAG	$p = 0.01$<br>–<br>$p < 0.05$
Anagnostopoulou *et al.* 2009 [3]	80	High fat meal (5.3 g protein, 24.75 g carbohydrate, 240 mg cholesterol, and 65.2 g fat—83.5% fat, 14.0% carbohydrates and 2.5% proteins)	S447X	Fasting / Postprandial TAG	No association
